# Diffuse Idiopathic Skeletal Hyperostosis, beyond the Axial Skeleton: Extra-spinal DISH

**DOI:** 10.31138/mjr.211024.bas

**Published:** 2025-03-31

**Authors:** Eleftherios Pelechas, Aliki I. Venetsanopoulou, Paraskevi V. Voulgari, Alexandros A. Drosos

**Affiliations:** Rheumatology Clinic, Department of Internal Medicine, Medical School, University of Ioannina, Ioannina, Greece

**Keywords:** DISH, extra-spinal DISH, enthesitis, tibial tuberosity

Diffuse idiopathic skeletal hyperostosis (DISH) is a very common disorder in patients with metabolic disorders. It affects mostly the axial skeleton, while peripheral DISH is not as common.^1,2^ We present a patient with mild symptoms and prominent and extensive manifestations of peripheral DISH.

A 68-year-old man presented to us complaining of stiffness affecting his knees and difficulty in walking that started 6 months ago. He denied psoriasis, colitis, visual disturbances, arthralgias, myalgias, dizziness, and ataxia. Past medical history was positive for metabolic syndrome (mS) and he was receiving atorvastatin, enalapril, and metformin. Family history was negative, and he was not nicotine-dependent. Clinical examination revealed a decreased range of motion in both shoulders. On knee examination, a crepitus sensation was present on palpation. On knee inspection, a large tibial protrusion on the tibial tuberosity was noted bilaterally, which was hard, firm and painless during palpation (**Figure 1**). Neurological examination was negative. Laboratory investigation showed only dyslipidaemia while the acute phase reactants and immunological tests were within normal limits or negative. Radiographs of the knees showed extensive degenerative changes with the presence of osteophytes, tricompartmental joint space narrowing (medial, lateral, and patellofemoral compartments), calcification and ossification of the superior and inferior patellar poles as well as ossified tibial tuberosities, with enthesopathy bilaterally (**Figures 2** and **3**). X-rays of the lumbar spine showed increased hypertrophic changes and excess bridging osteophytes between the vertebral bodies (**Figure 4**).

**Figure 1 (above, left).**
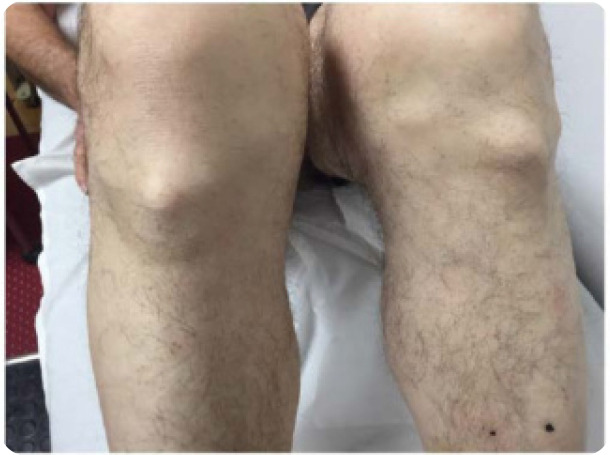
Large tibial protrusion on the tibial tuberosity is evident bilaterally which was hard, firm, and painless during palpation.

**Figure 2 (above, right).**
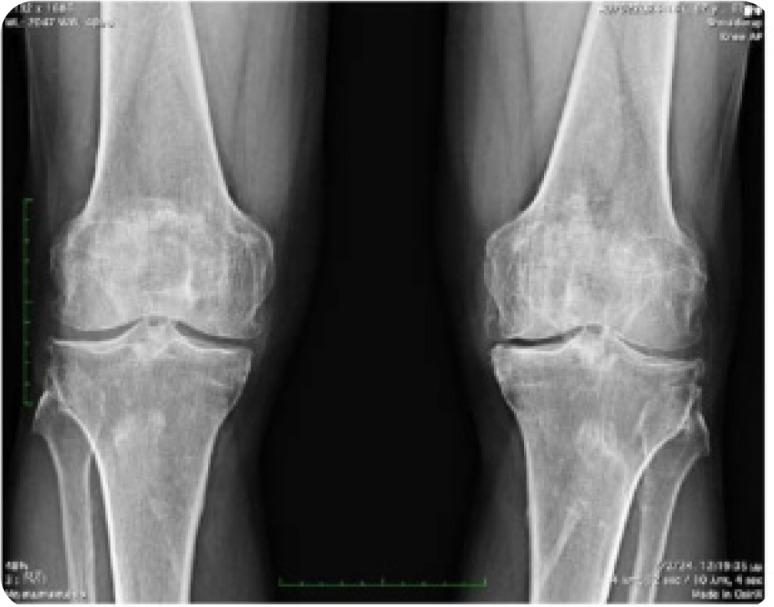
Radiographs of the knees showed extensive degenerative changes with the presence of osteophytes and tricompartmental joint space narrowing.

**Figure 3 (below).**
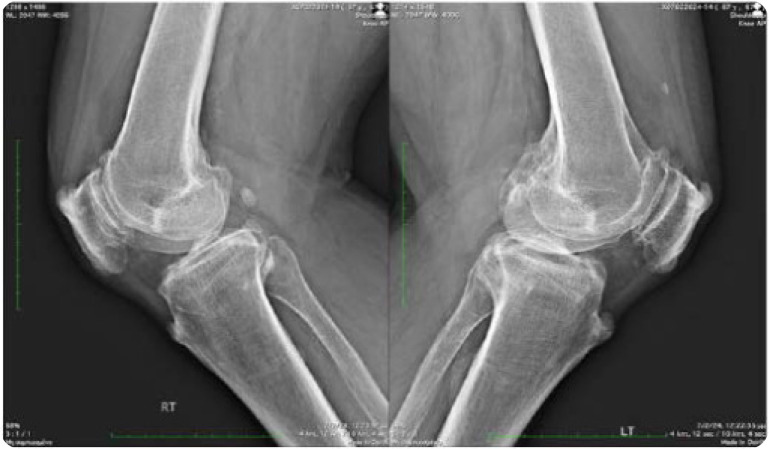
Lateral view of knees x-rays shows calcification and ossification of the superior and inferior patellar poles as well as ossified tibial tuberosities with enthesopathy bilaterally.

**Figure 4.**
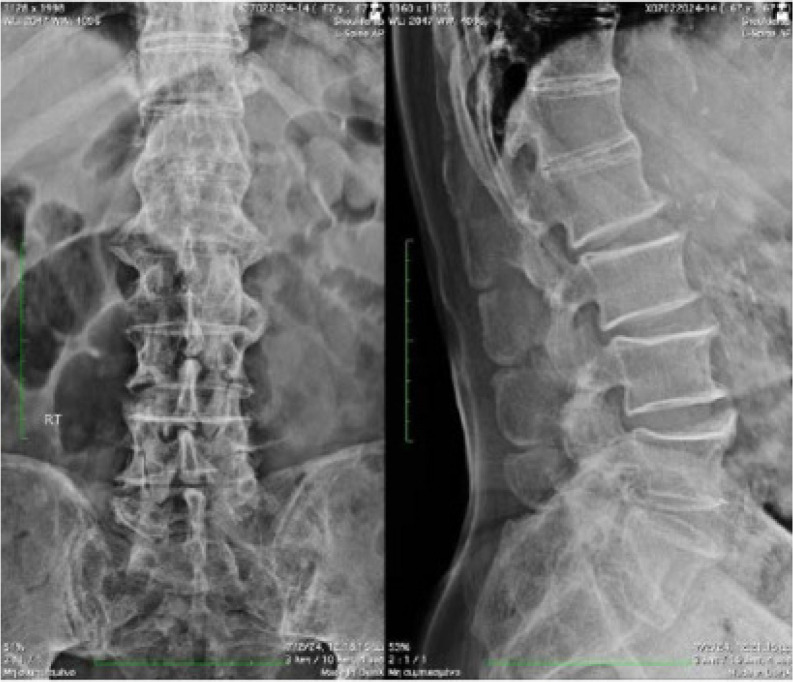
Lumbar spine x-rays show hypertrophic changes and excess bridging osteophytes between the vertebral bodies.

The above findings are characteristic for DISH, or Forestier’s disease, characterised by excessive calcification and ossification of periarticular soft tissues, entheses, ligaments and joints capsules. DISH is common in the elderly population and is associated with mS and other metabolic disorders.^1^ The classical site of involvement is the axial skeleton with linear new bone formation along the right anterolateral aspect of the thoracic spine.^2^ Extraspinal manifestations or peripheral involvement comprises joints, usually unaffected from primary osteoarthritis (OA), such as the carpal bones and metacarpophalangeal joints, but it can be a concomitant finding in OA patients.^3^ Another characteristic is the hypertrophic changes observed, compared to primary OA and the prominent enthesopathies of various sites adjacent to peripheral joints. In the majority of the patients, DISH is a radiological entity with no significant clinical implications, as in the case we presented above.^4^ In addition, rheumatologists should keep in mind that the majority of manifestations of the peripheral skeleton are not attributed to systemic autoimmune diseases but to degenerative and metabolic diseases.

## DECLARATION

This study was performed in accordance with the Helsinki Declaration of 1964 and its later amendments. All presented material is published after written consent of the patients, although sensitive data and personal details are not included in the publication.

## AUTHOR CONTRIBUTIONS

All authors have contributed equally for the production of the current manuscript. Pelechas E: drafting and editing, Venetsanopoulou AI: review and editing, Voulgari PV: review and editing, Drosos AA: captures the idea, final review. All authors have read and approved the final submitted version.

## FUNDING

None.

## CONFLICT OF INTEREST

None declared.
